# Sibling Disconnectedness, Sibship Size, and Cognition in Later Life

**DOI:** 10.1093/geroni/igaf122.2788

**Published:** 2025-12-31

**Authors:** Gina Lee, Jooyoung Kong, Deborah Carr, Michal Engelman

**Affiliations:** University of Wisconsin–Madison, Madison, Wisconsin, United States; University of Wisconsin–Madison, Madison, Wisconsin, United States; Boston University, Boston, Massachusetts, United States; University of Wisconsin-Madison, Madison, Wisconsin, United States

## Abstract

Prior research has established that social disconnectedness can negatively impact cognition in later life. However, sibling disconnectedness and its effect on cognitive change in older adults remain unexplored. We explore how sibling disconnectedness and sibship size interact to influence cognitive change in later life. Siblings are a potentially important source of social engagement, especially as older adults experience widowhood and deaths of friends. We used four waves of data from the Wisconsin Longitudinal Study (WLS; N = 6,468) collected in 1993, 2004, 2011, and 2020. The WLS tracked graduates of Wisconsin high schools in 1957 and one randomly selected sibling, into their late adulthood. Sibling disconnectedness was measured as binary indicators (W1-W3), defined as feeling “not at all close” to their selected sibling or having zero contact over the last 12 months. Global cognition (W2-W4) was assessed by averaging four standardized cognitive performance tests. A longitudinal linear mixed model was used. Results indicated that individuals disconnected from siblings in smaller sibships displayed an accelerated rate of cognitive decline. However, for individuals maintaining sibling connections, cognition levels did not differ across varying sibship sizes. Having more siblings appears cognitively beneficial for those experiencing sibling disconnection, potentially due to the support systems and mentally stimulating interactions with multiple network members. These findings underscore the importance of maintaining robust sibling ties, particularly for those with fewer siblings, in later life. Practitioners could leverage this knowledge to develop interventions/programs that promote family cohesion, particularly among siblings, and help mitigate cognitive decline among older adults.

